# Insight as a social identity process in the evolution of psychosocial functioning in the early phase of psychosis

**DOI:** 10.1017/S0033291716002506

**Published:** 2016-11-21

**Authors:** H. S. Klaas, A. Clémence, R. Marion-Veyron, J.-P. Antonietti, L. Alameda, P. Golay, P. Conus

**Affiliations:** 1 Swiss National Centre of Competence in Research LIVES, Life Course and Inequality Research Centre (LINES), Faculty of Social and Political Sciences, University of Lausanne, Switzerland; 2 Psychology Institute, Faculty of Social and Political Sciences, University of Lausanne, Lausanne, Switzerland; 3 Treatment and Early Intervention in Psychosis Program (TIPP), Service of General Psychiatry, Département de Psychiatrie Centre Hospitalier Universitaire Vaudois (CHUV), Université de Lausanne, Clinique de Cery, 1008 Prilly, Switzerland

**Keywords:** Early phase of psychosis, insight, schizophrenia, social functioning, social identity, TIPP

## Abstract

**Background:**

Awareness of illness (insight) has been found to have contradictory effects for different functional outcomes after the early course of psychosis. Whereas it is related to psychotic symptom reduction and medication adherence, it is also associated with increased depressive symptoms. In this line, the specific effects of insight on the evolution of functioning over time have not been identified, and social indicators, such as socio-occupational functioning have barely been considered. Drawing from social identity theory we investigated the impact of insight on the development of psychosocial outcomes and the interactions of these variables over time.

**Method:**

The participants, 240 patients in early phase of psychosis from the Treatment and Early Intervention in Psychosis Program (TIPP) of the University Hospital of Lausanne, Switzerland, were assessed at eight time points over 3 years. Cross-lagged panel analyses and multilevel analyses were conducted on socio-occupational and general functioning [Social and Occupational Functioning Assessment Scale (SOFAS) and Global Assessment of Functioning (GAF)] with insight, time and depressive symptoms as independent variables.

**Results:**

Results from multilevel analyses point to an overall positive impact of insight on psychosocial functioning, which increases over time. Yet the cross-lagged panel analysis did not reveal a systematic positive and causal effect of insight on SOFAS and GAF scores. Depressive symptoms seem only to be relevant in the beginning of the treatment process.

**Conclusions:**

Our results point to a complex process in which the positive impact of insight on psychosocial functioning increases over time, even when considering depressive symptoms. Future studies and treatment approaches should consider the procedural aspect of insight.

## Introduction

When comparing first-episode-psychosis patients to those suffering from other mental illnesses, the proportion of people showing no insight into their illness is especially high (Fennig *et al.* 1996; Conus *et al.* 2007; Saeedi *et al.* 2007). Lack of insight can be defined as an unawareness of the deficits, the consequences of the disorder and the need for treatment (Amador *et al.* 1991). Based on the literature, the impact of insight on different functional outcomes seems to be contradictory. On the one hand, awareness of illness is linked to treatment compliance, such as medication adherence and positive and negative symptom improvement (Mintz *et al.* 2004; McEvoy *et al.* 2006; Buchy *et al.* 2010). On the other hand, insight has been associated with increased depressive symptoms (Crumlish *et al.* 2005; McEvoy *et al.* 2006; Buchy *et al.* 2010; Cotton *et al.* 2012), especially at the beginning of the treatment process (Saeedi *et al.* 2007). Furthermore, insight into illness has been related to a decrease in quality of life (Kravetz *et al.* 2000) and self-esteem (Lysaker *et al.* 2007). Previous research has not yet disentangled these conflicting effects on functioning due to two main pitfalls: first, the covariation between insight and functional outcomes has not been thoroughly studied longitudinally, and second, a psychosocial perspective on the interaction between insight and remission from early psychosis has barely been considered in papers published so far.

Studies investigating the interaction between insight and the process of remission from early psychosis over time assessed patients either retrospectively (Cotton *et al.* 2009) or prospectively over a relatively short time-frame (Mintz *et al.* 2004; Parellada *et al.* 2009; Buchy *et al.* 2010; Capdevielle *et al.* 2013; O'Connor *et al.* 2013). Few studies followed patients up to 3 or 4 years either retrospectively (Gómez-de-Regil *et al.* 2010) or prospectively (Crumlish *et al.* 2005; Saeedi *et al.* 2007). Yet these studies assessed information only once a year and did not allow a detailed exploration of the evolution of functioning.

Furthermore, predominant models explaining a lack of insight consider the individual without taking the social context into account. While some explanatory models for lack of insight suggest a central role for internal mechanisms resulting directly from psychosis or resulting from cognitive deficits linked to the disorder (e.g. Henriksen & Parnas, 2014), others conceptualize it as a motivational defense mechanism (Thompson *et al.* 2001; for an overview, see Cooke *et al.* 2005).

When facing a first episode of psychosis, patients do not only need to cope with the symptoms of the disorder, but also with the emergence of a new identity and the possible loss of old ones; therefore, taking the perspective of social identity theory (Tajfel & Turner, 1986; Ellemers *et al.* 2002) – and its applications to health events such as coping with stroke (Haslam *et al.* 2008) or depression (Cruwys *et al.* 2013) – seems highly relevant when exploring this domain. Indeed, in the early phase of psychosis patients have to integrate a new self-definition and must do so within a social context in which schizophrenia is stigmatized (Lauber *et al.* 2000; Thornicroft *et al.* 2009); an acknowledgement of the ‘schizophrenic’ identity therefore threatens their self-esteem. The phenomenon of social categorization implies that the patient will be perceived and treated differently than before by others, which may explain why he or she is likely to be torn between acknowledgment and refusal of the schizophrenic identity. The development of functioning is therefore not limited to the remission of clinical symptoms, and should rather, as recently suggested, be seen ‘primarily (as) a process of transforming identity’ (Williams, 2008, p. 246; see also Haslam *et al.* 2009).

We argue that this identity transformation, which is central for the evolution of functional outcomes, can best be observed when considering the evolution of social and occupational functioning. Surprisingly, even if the social functioning in everyday life represents a very important domain of remission from schizophrenia as it is an important dimension of social integration (Rinaldi *et al.* 2010), longitudinal research on insight and early psychosis has so far focused predominantly on its link to clinical symptoms (Buchy *et al.* 2010; O'Connor *et al.* 2013). When considering research investigating established schizophrenia, there is some cross-sectional (Karow *et al.* 2008; Giugiario *et al.* 2012) and longitudinal evidence (Lincoln *et al.* 2007; Mohamed *et al.* 2009; Parellada *et al.* 2009) that an improvement in insight is related to an improvement of social or occupational functioning. One recent study found an increasingly positive association of insight and social functioning over the period of 1 year in involuntarily admitted patients with schizophrenia (van Baars *et al.* 2013). These findings indicate a positive correlation between insight and social integration, but they do not provide sufficient information regarding both the evolution and the causal direction of this relation in the early phase of psychosis.

The present study tries to address the mentioned research gaps in the context of the prospective 3-year follow-up of early psychosis patients with two measurement points per year. Based on social identity theory, we hypothesized that the interactions of insight and psychosocial functioning over time would depend on the process in which a person acknowledges or not the mental disorder and therefore the social identity of a ‘schizophrenic person’ (Williams, 2008). Following this reasoning, a recognition of the illness (insight) and hence the perception of the negative social label attached to it should lead in the beginning of the evolution of functioning to an increase in depressive symptoms. In order to recover or maintain a positive self-esteem and positive relationships with others (Tajfel & Turner, 1986; Baumeister & Leary, 1995), the patient might then be led to resist the stigmatized self-definition (no insight), which should be associated with decreased depressive symptoms. Maintaining denial, however, is often complicated when facing the consequences of the illness. Furthermore, over time, recognizing the illness could also have positive effects, considering that the relationship with caregivers often depends on the patients acknowledging their difficulties, which in turn fosters good social interactions within the treatment context, promotes a better self-esteem and enhances the protection against stigma and negative stereotyping (Crocker & Major, 1989; Camp *et al.* 2002; Crabtree *et al.* 2010).

With this in mind, the aims of this study were to explore the pattern of interactions between insight, both self-esteem and depressive symptoms, and social functioning over the 3 first years of treatment in a sample of early phase of psychosis patients in the frame of a specialized early intervention program. Hypotheses were that

(1) The level of insight would be correlated with the level of functional outcome, and this positive correlation would increase with time.
(2) The correlation of depressive symptoms with insight would decrease while the correlation of self-esteem and insight would increase with time.
(3) Change of insight between two time points would cause a corresponding change of functional outcome. That is, insight at time *X* should have a positive effect on functional outcome at time *X* + 1.

## Method

### Procedure and participants

Treatment and early Intervention in Psychosis Program (TIPP), a specialized early psychosis program, was launched in 2004 at the Department of Psychiatry CHUV, in Lausanne, Switzerland, and the concept and study methods have been described elsewhere in details (Baumann *et al.* 2013). Entry criteria to the program are: (i) age between 18 and 35 years; (ii) residence in the catchment area; (iii) meeting threshold criteria for psychosis, as defined by the ‘Psychosis threshold’ subscale of the Comprehensive Assessment of At Risk Mental States (CAARMS; Yung *et al.* 2005) scale. Patients are referred to other treatment programs if they have been taking antipsychotic medication for more than a total of 6 months, have psychosis related to intoxication or organic brain disease, or have an intelligence quotient below 70.

A specially designed questionnaire (the TIPP Initial Assessment Tool: TIAT) is completed for all patients enrolled in the program by case managers who, in the frame of a caseload of 30 patients, have up to 100 contacts with patients during the 3 years of treatment. It allows assessment of demographic characteristics, past medical history, exposure to life events as well as symptoms and functioning. It is completed on the basis of information gathered with patients and their family over the first few weeks of treatment and can be updated during follow-up if new information emerges. Follow-up assessments exploring various aspects of treatment and co-morbidities as well as evolution of psychopathology and functional level are conducted by trained research psychologists for scale based assessment (with assessment of inter-rater reliability) and by case managers (for objective measures such as employment and living conditions for example) after 2, 6, 12, 18, 24, 30 and 36 months in treatment. In particular, substance abuse diagnosis was assessed at each time point and rated on the basis of DSM-IV criteria (APA, 2000). The Research and Ethics Committee of the Faculty of Biology and Medicine of Lausanne University granted access to TIPP clinical data for research purposes.

The present study was based on the 240 patients who had been enrolled in the program and who had been in treatment for 36 months at the time of the study. They were between 17 and 37 years old (mean = 24.0, s.d. = 4.8) when entering the program, and lived in the urban area of Lausanne, one of the biggest cities in Switzerland (about 300000 inhabitants). The city is economically well developed, especially in the fields of health, research, education, social services and culture, and also banking, insurance and tourism. More than 40% of people are immigrants, most of them coming from European countries. Compared to the population of this generation in Lausanne, men (*n* = 166, 69% of the sample) and immigrants (*n* = 155, 65%) were overrepresented. Regarding the family situation, 83% of the patients were single, and 42% lived with their parents. The level of education, measured by years of school, corresponded to the achievement of the compulsory school, the lowest level in Switzerland (mean = 9.5, s.d. = 1.3), and 66% had no professional or educational activity when they entered the program.

### Diagnostic assessment

In the TIPP program, diagnosis is the result of an expert consensus and is based on the following elements: (1) Diagnosis reported by a treating psychiatrist in all medical documents and at the end of any hospitalization; (2) Longitudinal assessment by clinical case managers over the 3 years of treatment. The consensus diagnosis procedure is realized by a senior psychiatrist and the senior psychologist who is in charge of scale-based assessment over the treatment period. They both review the entire file once after 18 months and again after 36 months or at the end of treatment and conduct a diagnostic process based on DSM-IV criteria (APA, 1994) discussing any unclear issue with the clinical case manager. In this paper, only the final diagnosis, defined at the end of the TIPP treatment period, is considered.

### Measures

*Socio-demographics and family characteristics* were collected at entry into the program, as was the past psychiatric history of the patients.

*Insight* was assessed by judgment of the case manager based on observation and conversation with the respective patient (0, absence of insight; 1, partial insight; 2, full insight). Full insight meant awareness of the illness and the necessity of treatment. Partial insight meant insight into the illness, but no awareness of the consequences and the necessity of treatment.

*Psychosocial functioning* was assessed by the Social and Occupational Functioning Assessment Scale (SOFAS; APA, 1994) ranging on a 1–100 scale, with 100 indicating a high level of functioning in a broad variety of activities without physical and psychic alterations in social, professional or academic relations and performances, and by the Global Assessment of Functioning Scale (GAF; Jones *et al.* 1995) ranging on a 1–100 scale, with 100 indicating a high level of functioning in a broad variety of activities going along with social functioning, the ability to cope with problems and the absence of symptoms. A self-rated construct of *satisfaction with life and self-esteem* was assessed by the 26 items of the World Health Organization Quality Of Life assessment scale (WHOQOL; The WHOQOL Group, 1995) based on 5-point Likert scales ranging from 1 (low satisfaction) to 5 (high satisfaction). *Medication adherence* for the past 2 months was assessed at each time point on a scale ranging from 1 (no adherence, adherence <25% of the time) to 3 (full adherence, adherence >75% of the time). A psychologist completed the Positive and Negative Syndrome Scale for Schizophrenia (PANSS; Kay *et al.* 1987) and the Brief Psychiatric Rating Scale (BPRS; Overall & Gorham, 1962) for half of the patients. PANSS, BPRS, medication adherence and WHOQOL were collected first at the second time point of the study. *Depressive symptoms* were assessed by averaging two items, one from the PANSS (G6: Depression, including feelings of sadness, discouragement, helplessness, and pessimism) and one from the BPRS (BPRS3: Depression, including sadness, unhappiness, anhedonia and preoccupation with depressing topics, hopelessness, loss of self-esteem), which were highly correlated at each time point (0.91 < *r* < 0.95).

### Statistical analysis

To test the first hypotheses, a multilevel longitudinal analysis was conducted in order to take into account the variations at the time and individual levels. A first analysis was conducted, with time as level 1 and individuals as level 2, to verify if insight exerted a main effect and moderated the time effect on the evolution of SOFAS and GAF. Similar analyses were realized on WHOQOL and depressive symptoms. Analyses were conducted with MLwiN.2 software by using the model of iterative generalized least squares (Kreft & De Leeuw, 1998; Rasbah *et al.* 2009).

The last hypothesis about the causal effect of insight on SOFAS and GAF at each time point was tested by using a cross-lagged panel analysis with AMOS v. 22 software www-03.ibm.com/software/products/fr/spss-amos and the lavaan procedure of R (www.r-project.org). We began by comparing a fully reciprocal model with a basic model without influence of both variables (autoregressive model), and tested the fit of the data of the former two models when insight and respectively SOFAS and GAF were successively the source or the target of influence.

Preliminary analyses were conducted on missing values to evaluate the influence of initial measures of the main variables (insight, SOFAS, GAF) on the attrition. Table 1 provides the breakdown of the participants included in the study at each time point for each variable. The missing values increase from 4.6% to 28.8% for insight, 5.4% to 26.7% for SOFAS and 8.8% to 26.7% for GAF. To check if the participants lost during the study differ for the three measures, we compared their scores when they entered the treatment program with the scores of the remaining participants. The comparisons revealed no statistically significant differences between the means of both groups on insight (mean_miss_ = 0.98, s.d. = 0.70; mean_rem_ = 0.79, s.d. = 0.71; *t*_227_ = 1.90, *p* = 0.06), SOFAS (mean_miss_ = 39.64, s.d. = 16.01; mean_rem_ = 37.65, s.d. = 15.84; *t*_225_ < 1) and GAF (mean_miss_ = 37.05, s.d. = 15.10; mean_rem_ = 34.59, s.d. = 15.67; *t*_217_ = 1.05, *p* = 0.30). Following these results, we used the maximum likelihood method (available in AMOS) to estimate the missing values for doing the cross-lagged panel analyses. In the multilevel framework all existing data were used in the estimation of the models. Then, the multilevel analyses were only conducted on the available observations.

**Table 1.** Evolution of insight, medication adherence, SOFAS, GAF, WHOQOL and depressive symptoms during treatment

Time points (month)	Insight mean (s.d.), *n*	Medication adherence mean (s.d.), *n*	SOFAS mean (s.d.), *n*	GAF mean (s.d.), *n*	WHOQOL mean (s.d.), *n*	Depressive symptoms mean (s.d.), *n*
0	0.84 (0.71) 229	–	38.21 (15.88) 227	35.26 (15.52) 219	–	–
2	1.14 (0.68) 213	1.50 (0.76) 202	49.95 (15.62) 217	47.12 (16.33) 217	3.32 (0.60) 86	3.17 (1.62) 112
6	1.33 (0.64) 203	1.45 (0.77) 195	55.39 (14.65) 210	53.33 (15.15) 212	3.40 (0.55) 94	2.87 (1.45) 135
12	1.38 (0.66) 195	1.49 (0.76) 180	57.28 (15.37) 206	55.85 (15.77) 207	3.45 (0.61) 92	2.78 (1.36) 121
18	1.42 (0.64) 179	1.49 (0.81) 166	58.26 (15.04) 192	56.50 (15.30) 192	3.60 (0.60) 101	2.63 (1.46) 122
24	1.40 (0.66) 175	1.56 (0.77) 167	57.24 (15.86) 186	55.78 (16.35) 185	3.55 (0.58) 95	2.44 (1.32) 121
30	1.46 (0.64) 168	1.57 (0.76) 170	58.23 (15.17) 174	57.00 (16.17) 174	3.64 (0.61) 91	2.19 (1.25) 111
36	1.44 (0.64) 171	1.57 (0.76) 171	58.94 (15.95) 176	57.10 (17.05) 176	3.61 (0.54) 81	2.31 (1.29) 94
Scale	0–2	1–3	1–100	1–100	1–5	1–5

SOFAS, Social and Occupational Functioning Assessment Scale; GAF, Global Assessment of Functioning Scale; WHOQOL, World Health Organization Quality Of Life assessment scale.

## Results

When entering the program, patients presented a marked impairment of level of SOFAS (mean = 38.2, s.d. = 15.9) and GAF (mean = 35.3, s.d. = 15.5). Insight was positively correlated with SOFAS (*r* = 0.33, *p* < 0.001) and GAF (*r* = 0.35, *p* < 0.001). The latter two measures were highly correlated (*r* = 0.90, *p* < 0.001). Age was slightly correlated with insight (*r* = 0.14, *p* = 0.041). There was no correlation between socio-demographic characteristics (sex, level of education, socio-professional origin, migration) and the three measures (SOFAS, GAF, insight).

**Fig. 1.**
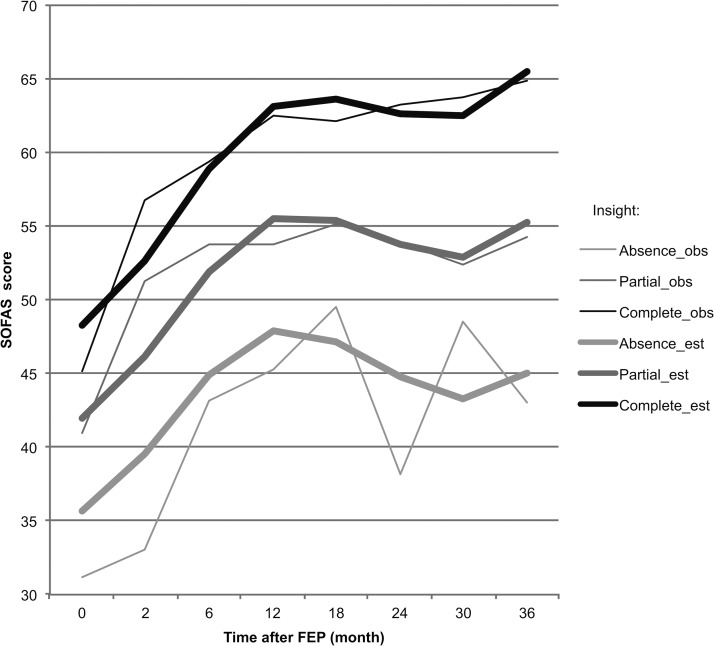
Evolution of estimated and observed SOFAS scores according to level of insight during the treatment. Insight: obs, SOFAS observed score; est, SOFAS estimated score (multilevel analysis).

The improvement of SOFAS and GAF after treatment initiation was followed by a collapse after 1 year, and a small improvement at the end of the observation (Table 1 and Fig. 1). There was a clear increase in insight, whereas medication adherence appeared to be more stable in time. The self-reported global self-esteem and satisfaction with life (WHOQOL) was high in comparison to the other measures and showed a slight improvement. In addition, the depressive symptoms diminished over time. In order to validate our insight measure for each time point of the study, we used one item of the PANSS (G12: lack of judgment and awareness), which has been used to validate insight in previous studies (e.g. Buchy *et al.* 2010). The item was highly correlated with insight at each time point (−0.56 < *r* < −0.40, *p* < 0.001). Insight was also correlated, but in a less systematic way, with medication adherence (0.25 < *r* < 0.56, *p* < 0.001). Depressive symptoms were positively correlated with insight 2 months after the beginning of the treatment (*r* = 0.21, *p* = 0.033), but this positive association disappeared subsequently (*r* = 0.09 after 6 months and *r* = −0.03 after 3 years).

**Fig. 2.**
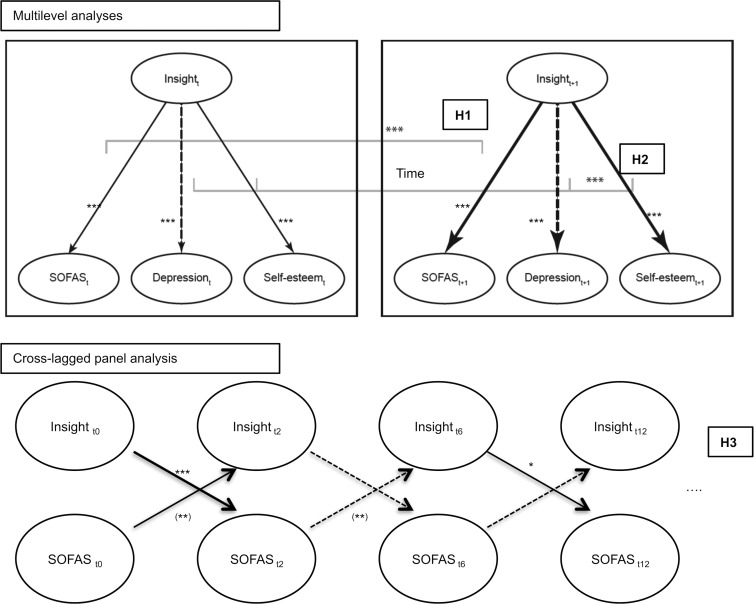
Schematic representation of the estimated relations between Insight, SOFAS, depressive symptoms and Self-esteem obtained from the multilevel and cross-lagged panel analyses. Continuous lines indicate a positive coefficient and dotted lines indicate a negative coefficient: *** *p* < 0.001; ** *p* < 0.01; * *p* < 0.05. Subscripts designate time points. H1, H2, H3 refer to the hypotheses: H1, positive effect of insight on SOFAS would increase with time; H2, negative effect of insight on depressive symptoms and positive effect of insight on self-esteem would increase with time; H3, insight at *t*_*x*_ would have a positive effect on SOFAS at *t*_*x*+1_.

A schematic presentation of the results obtained by the multilevel analyses can be found in Fig. 2. The multilevel analysis on SOFAS and GAF scores revealed that adding the main effect of insight and the interaction effect of insight and time (Table 2, models 2) clearly improved the models taking into account only the effects of time (models 1).

**Table 2.** Estimates and standard errors of the multilevel longitudinal modeling of the evolution of SOFAS and GAF with insight

	SOFAS			GAF		
	Model 0	Model 1	Model 2	Model 0	Model 1	Model 2
Fixed effects						
Intercept	53.1328 (0.8149)	41.0592 (0.9599)	35.6630 (1.1896)	51.3587 (0.8281)	38.2530 (0.9899)	31.7478 (1.1894)
Level 2 (individuals)						
Insight			6.3089 (0.8316)			7.4195 (0.8378)
Insight × time			0.1086 (0.0332)			0.0996 (0.0348)
Level 1 (time)						
Time (linear)		2.9038 (0.1909)	2.1879 (0.1802)		3.0465 (0.2005)	2.2749 (0.1894)
Time (quadratic)		−0.1460 (0.0134)	−0.1186 (0.0126)		−0.1495 (0.0141)	−0.1195 (0.0132)
Time (cubic)		0.0022 (0.0003)	0.0018 (0.0002)		0.0022 (0.0003)	0.0018 (0.0002)
Random effects						
Individual variance	129.1936 (14.4640)	134.3735 (14.1748)	143.9920 (23.076)	131.1189 (14.8826)	138.1630 (14.6487)	131.0208 (22.4000)
Insight slope variance			37.2646 (8.7981)			29.7610 (8.4465)
Insight × intercept covariance			−38.6373 (12.4359)			−29.4393 (11.9537)
Time variance	159.5469 (6.3370)	110.9210 (4.4073)	87.9493 (3.7163)	177.7199 (7.0431)	120.9925 (4.7789)	97.23 338 (4.1016)
Deviance	12316.04	11847.24	11605.59	12419.59	11926.98	11670.49
Observations	1504	1504	1504	1499	1499	1499

SOFAS, Social and Occupational Functioning Assessment Scale; GAF, Global Assessment of Functioning Scale.

All effects are statistically significant at *p* < 0.001 (Wald χ^2^_1_ test).

Both models confirmed an important increase in functioning in the beginning of the treatment and an enhanced tendency of improvement in the end of the time-frame of observation (Fig. 1). They also revealed a slight decrease in functioning in the middle of the process. The significant random effect of insight at the individual level shows that there are important individual differences, which depend in particular on the level of initial psychosocial and functioning resources: The increase in functioning was inversely associated with the initial levels of SOFAS and GAF.

The depressive symptoms starting from time point two were then added into the model as there were no measures for time point zero. The multilevel model did hence not include time point zero, and the number of valid observations diminished (*n* = 743). Nevertheless, results confirm the preceding observation except for the disappearance of the significant interaction between insight and time due to the exclusion of the first time point. Depressive symptoms exerted a significant principal and negative effect (SOFAS: *β* = −1.995, s.e. = 0.303, *p* < 0.001; GAF: *β* = −2.327, s.e. = 0.319, *p* < 0.001), but did not interact with time nor decreased or moderated the effect of insight. Moreover, the principal effect of insight completely suppressed the moderate positive effect of medication adherence on both psychosocial and functioning measures.

Multilevel analyses were then conducted on the depressive symptoms and the WHOQOL, as a self-reported measure of self-esteem and life satisfaction. The results (Table 3) show an interaction effect of insight and time on the depressive symptoms (model 2), which suppressed the main effect of time (model 1). Depressive symptoms decreased more in time with a high degree of insight. We observed a cumulative linear and quadratic effect of time and an interaction effect of insight and time on the WHOQOL. Patients evaluated their self-esteem independently of their insight at the beginning of the treatment, but the improvement in time of the WHOQOL was then positively correlated to insight.

**Table 3.** Estimates and standard errors of the multilevel longitudinal modeling of the evolution of the depressive symptoms and the WHOQOL from second to last measurement point with insight

	Depressive symptoms			WHOQOL		
	Model 0	Model 1	Model 2	Model 0	Model 1	Model 2
Fixed effects						
Intercept	2.6388 (0.0765)***	3.1037 (0.0978)***	3.0020 (0.0955)***	3.5049 (0.0406)***	3.2765 (0.0571)***	3.2822 (0.0589)***
Level 2 (individuals)						
Insight			–			–
Insight × time			−0.0132 (0.0020)***			0.0056 (0.0017)***
Level 1 (time)						
Time (linear)		−0.0274 (0.0037)***	–		0.0224 (0.0055)***	0.0150 (0.0059)**
Time (quadratic)		–	–		−0.0004 (0.0001)**	−0.0004 (0.0001)**
Random effects						
Individual variance	0.8426 (0.1192)	0.8401 (0.1164)	0.9547 (0.1292)	0.2208 (0.0297)	0.2235 (0.0296)	0.2177 (0.0289)
Time variance	1.2075 (0.0689)	1.1160 (0.0637)	1.0436 (0.0626)	0.1391 (0.0093)	0.1285 (0.0086)	0.1267 (0.0084)
Deviance	2728.73	2674.46	2451.92	828.94	790.63	779.55
Observations	816	816	816	613	613	613

WHOQOL, World Health Organization Quality Of Life assessment scale.

*** *p* < 0.001; ** *p* < 0.01 (Wald χ^2^_1_ test).

A schematic presentation of the results obtained by the cross-lagged panel analysis can be found in Fig. 2. Our last hypothesis concerned the causal direction of the relations between insight and the two measures of psychosocial functioning. The results revealed that the cross-lagged SOFAS and insight model fitted the data better than the basic autoregressive model, respectively (χ^2^_72_ = 89.91, *p* = 0.084, CFI = 0.991, RMSEA = 0.032, 90% CI 0.000–0.051; χ^2^_86_ = 144.24, *p* < 0.001, CFI = 0.969, RMSEA = 0.053, 90% CI 0.038–0.068; Δχ^2^_14_ = 54.33, *p* < 0.001). The two models predicting a unilateral effect (of insight on SOFAS or the reverse) had worse fit than the cross-lagged model (both models: Δχ^2^_7_ > 25.00, *p* < 0.01). The regression coefficients indicated a more complex pattern of relations between SOFAS and insight than the expected one (Table 4).

**Table 4.** Regression weights and standard errors of the cross-lagged models between insight and respectively SOFAS and GAF

Time points (*t*)	Regression weights (s.e.)			
	Insight_*t*−1_ 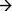 SOFAS*_t_*	SOFAS_*t*−1_ 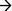 Insight*_t_*	Insight_*t*-1_ 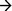 GAF*_t_*	GAF_*t*-1_ 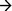 Insight_*t*_
2	4.133 (1.277)***	0.006 (0.002)**	4.056 (1.257)***	0.006 (0.003)*
6	−0.970 (1.555)	−0.010 (0.004)**	−0.176 (1.680)	−0.009 (0.004)*
12	2.905 (1.211)*	−0.002 (0.003)	2.243 (1.422)	−0.004 (0.003)
18	−2.277 (1.303)	−0.005 (0.003)	−1.473 (1.430)	−0.003 (0.003)
24	3.102 (1.359)*	0.003 (0.003)	2.043 (1.503)	0.004 (0.003)
30	−3.328 (1.540)*	−0.006 (0.004)	−2.958 (1.688)	−0.006 (0.004)
36	2.118 (1.388)	−0.003 (0.003)	0.520 (1.652)	−0.003 (0.003)

SOFAS, Social and Occupational Functioning Assessment Scale; GAF, Global Assessment of Functioning Scale.

*** *p* < 0.001; ** *p* < 0.01; * *p* < 0.05.

There was a strong and positive mutual influence at the beginning of the treatment. The level of SOFAS had then a significant negative effect on the consecutive level of insight and a slightly negative influence in the first year after the early phase of psychosis and in the end of the treatment. The effects of insight alternated between positive and negative influences on the consecutive level of SOFAS, even if the positive effects were stronger. Finally, a more parsimonious model including only the significant paths (four of insight on SOFAS and two in the other direction) did not differ from the complete cross-lagged model (χ^2^_78_ = 100.96, *p* = 0.041, CFI = 0.988; RMSEA = 0.035, 90% CI 0.008–0.053; Δχ^2^_6_ = 11.05, *p* > 0.10).

The analysis conducted on the cross-lagged GAF and insight model offered comparable results. First, the cross-lagged model fitted the data better than the autoregressive one, respectively (χ^2^_72_ = 86.14, *p* = 0.122, CFI = 0.991; RMSEA = 0.029, 90% CI 0.000–0.049; χ^2^_86_ = 130.44, *p* = 0.001, CFI = 0.992, RMSEA = 0.047, 90% CI 0.029–0.062; Δχ^2^_14_ = 44.30, *p* < 0.001). Then, both unilateral effects models significantly differed from the cross-lagged model, but the unilateral effect of insight on GAF showed a worse fit (Δχ^2^_7_ = 24.32, *p* < 0.01) than the reverse one (Δχ^2^_7_ = 17.57, *p* < 0.05). As in the preceding analysis, we observed a strong and positive mutual influence in the beginning of the treatment, followed by a significant negative effect of GAF on the consecutive insight. Then, no effect reached a statistical significant influence (Table 4). Yet, a parsimonious model including only the three significant effects (two of GAF on insight and one in the reverse direction) was worse than the complete cross-lagged model (χ^2^_83_ = 106.34, *p* = 0.043, CFI = 0.987, RMSEA = 0.034, 90% CI 0.007–0.052; Δχ^2^_11_ = 20.20, *p* < 0.05), which suggests that one should rather consider the global pattern.

These results showed a strong initial causal and reciprocal influence, but then the paths indicated contrasting positive and negative effects of insight on SOFAS and GAF, while in return SOFAS and GAF tend to diminish the level of insight.

## Discussion

The present study investigated the modulating effect of insight in the early phase of psychosis on the evolution of psychosocial functioning over 3 years. It filled existing research gaps by following patients thoroughly over time within a social identity perspective. Our results point to the importance of insight for the development of psychosocial outcomes in the early course of psychosis and complement a few previous findings obtained with patients suffering from established schizophrenia (van Baars *et al.* 2013). As expected, they demonstrate an evolving positive relation of insight over the initial phase of treatment for emerging psychosis, which remains stable when controlling for depressive symptoms. Furthermore, they also show a positive effect of insight with time on a measure of self-reported satisfaction with life and self-esteem and a negative effect on depressive symptoms. Depressive symptoms seem to be a drawback in the beginning of the process, which is in line with previous studies for shorter time-frames (Mintz *et al.* 2004; Saeedi *et al.* 2007). Our hypothesis of a positive causal impact of insight on social functioning could not be systematically confirmed. The cross-lagged analysis revealed a strong reciprocal influence between insight and the two measures of psychosocial functioning at the beginning of the treatment, followed by a negative effect of psychosocial functioning on insight in a second phase. After 1 year, there are only slight effects of insight, which alternatively increase or decrease the functional outcomes. This pattern of results points to a more complex interaction between variations in adherence to an illness identity and variations in the evolution of psychosocial functioning.

Even if our study did not directly assess identity variables, the findings suggest that identity processes intervene in the regulation of psychosocial functioning. First, patients develop a more positive illness identity during the treatment, as evidenced by the increasing positive relation between insight and self-reports of life satisfaction and self-esteem and the decreasing negative relation between insight and depressive symptoms during the process. Second, the positive relation of insight and social and occupational functioning increased over time, and patients with full insight follow a more stable recuperation curve after 1 year than those with no insight. This evolution can easily be articulated with the adherence to an illness identity associated with the enhancement of self-esteem, which favors in return the recuperation of psychosocial functioning (Haslam *et al.* 2009). Third, considering insight and identity processes as a socio-cognitive conflict of identification within the development of a new social self-definition, the complexity of the interaction of the causal links between insight and functioning outcomes might reflect the actual conflict of identification. This is supported by the intrapersonal variability we find in insight over time. In the same way, the negative relationships of SOFAS and GAF on insight after the important functional improvement at the beginning of the treatment can be explained when considering that a fast gain in psychosocial resources could also lead patients to revise their acknowledgment of the diagnosis and deny the endorsement of a schizophrenic identity. Hence, the bidirectional positive and negative interactions could reflect the process in which psychosocial functioning can either stress a lack of insight, or, on the other hand, enforce the development of insight (Williams, 2008). Then, the situation at treatment onset shows an interdependency of insight and identity-related variables. In this context, it would be interesting for future studies to investigate more directly the identity process by using specific measures of identification and by differentiating between aspects focusing on activities and aspects focusing on relations in psychosocial functioning.

Some limitations of our study need to be mentioned. First, insight, SOFAS and GAF were evaluated by the same case managers who could have inferred the level of insight from the level of the psychosocial functioning. However, the probability of such a bias appears weak. Case managers had to integrate information from third parties in order to evaluate the work and family environment of the patient and did therefore not only rely on their subjective perception. Furthermore, the level of insight is validated by the correlations with a pertinent item of the PANSS, which was rated by a psychologist, and the results of the effects of insight on the self-rated WHOQOL confirm those obtained with SOFAS and GAF. Second, insight was conceptualized as a holistic measure in this study comprising the awareness of illness as well as the acceptance of treatment while recent studies suggest insight is multidimensional and should be assessed as such (Tranulis *et al.* 2008; Capdevielle *et al.* 2013). This is due to the fact that the current study is based on data collected prospectively in the frame of a cohort study where the initial aim was not restricted to the study of insight and its correlation with outcomes. Besides, in the literature, a correlation between psychosocial outcomes and insight was mainly found regarding the self-reflection related cognitive component of insight (Raffard *et al.* 2013), a dimension which we did not include in the current study. Given numerous conceptualizations and measurements of insight (self-rated, observed or by interview; one or several dimensions; see also Dam, 2006), future research should vary insight measures and compare them to each other with regard to their interactions with psychosocial functioning. Finally, our study encountered a common limitation in clinical repeated measures with high missing and attrition data rates. Yet, a comparison between valid and missing patient data at each time point revealed no significant differences regarding the measures we used in this study.

## Conclusions

Our findings show the central role which insight plays for the evolution of psychosocial functional outcomes in the early phase of psychosis. Future studies should include direct identity measures in order to disentangle the complex interactions revealed by some results of the study. This extends to treatment approaches specifically aiming at the improvement of insight, which has proven moderate effects so far (Pijnenborg *et al.* 2013). During the treatment of early phase of psychosis, one should consider identity processes of the patients and, if it is possible, accept that these take time. An inclusion of the social identity perspective could add significant value to both research and treatment perspectives in early course of psychosis and schizophrenia.

## References

[ref1] Amador XF, Strauss DH, Yale SA, Gorman JM (1991). Awareness of illness in schizophrenia. Schizophrenia Bulletin 17, 113–132. 2047782 10.1093/schbul/17.1.113

[ref2] APA (1994). Diagnostic and Statistical Manual of Mental Disorders, 4th edn American Psychiatric Association: Washington, DC.

[ref3] APA (2000). Diagnostic and Statistical Manual of Mental Disorders, 4th edn (text revision). American Psychiatric Association: Washington, DC.

[ref4] Baumann PS, Crespi S, Marion-Veyron R, Solida A, Thonney J, Favrod J, Bonsack C, Do KQ, Conus P (2013). Treatment and early intervention in psychosis program (TIPP-Lausanne): implementation of an early intervention programme for psychosis in Switzerland. Early Intervention in Psychiatry 7, 322–328. 23445318 10.1111/eip.12037

[ref5] Baumeister RF, Leary MR (1995). The need to belong: desire for interpersonal attachments as a fundamental human motivation. Psychological Bulletin 117, 497–529. 7777651

[ref6] Buchy L, Bodnar M, Malla A, Joober R, Lepage M (2010). A 12-month outcome study of insight and symptom change in first-episode psychosis. Early Intervention in Psychiatry 4, 79–88. 20199483 10.1111/j.1751-7893.2010.00166.x

[ref7] Camp DL, Finlay WML, Lyons E (2002). Is low self-esteem an inevitable consequence of stigma? An example from women with chronic mental health problems. Social Science & Medicine 55, 823–834. 12190273 10.1016/s0277-9536(01)00205-2

[ref8] Capdevielle D, Norton J, Jaussent I, Prud'homme C, Raffard S, Gelly F, Boulenger J-P, Ritchie K (2013). A multi-dimensional approach to insight and its evolution in first-episode psychosis: a 1-year outcome naturalistic study. Psychiatry Research 210, 835–841. 24126187 10.1016/j.psychres.2013.09.020

[ref9] Conus P, Cotton S, Schimmelmann BG, McGorry PD, Lambert M (2007). The first-episode psychosis outcome study: premorbid and baseline characteristics of an epidemiological cohort of 661 first-episode psychosis patients. Early Intervention in Psychiatry 1, 191–200.

[ref10] Cooke MA, Peters ER, Kuipers E, Kumari V (2005). Disease, deficit or denial? Models of poor insight in psychosis. Acta Psychiatrica Scandinavica 112, 4–17. 15952940 10.1111/j.1600-0447.2005.00537.x

[ref11] Cotton SM, Lambert M, Schimmelmann BG, Foley DL, Morley KI, McGorry PD, Conus P (2009). Gender differences in premorbid, entry, treatment, and outcome characteristics in a treated epidemiological sample of 661 patients with first episode psychosis. Schizophrenia Research 114, 17–24. 19635660 10.1016/j.schres.2009.07.002

[ref12] Cotton SM, Lambert M, Schimmelmann BG, Mackinnon A, Gleeson JFM, Berk M, Hides L, Chanen A, McGorry PD, Conus P (2012). Depressive symptoms in first episode schizophrenia spectrum disorder. Schizophrenia Research 134, 20–26. 21937197 10.1016/j.schres.2011.08.018

[ref13] Crabtree JW, Haslam SA, Postmes T, Haslam C (2010). Mental health support groups, stigma, and self-esteem: positive and negative implications of group identification. Journal of Social Issues 66, 553–569.

[ref14] Crocker J, Major B (1989). Social stigma and self-esteem: the self-protective properties of stigma. Psychological Review 96, 608–630.

[ref15] Crumlish N, Whitty P, Kamali M, Clarke M, Browne S, McTigue O, Lane A, Kinsella A, Larkin C, O'Callaghan E (2005). Early insight predicts depression and attempted suicide after 4 years in first-episode schizophrenia and schizophreniform disorder. Acta Psychiatrica Scandinavica 112, 449–455. 16279874 10.1111/j.1600-0447.2005.00620.x

[ref16] Cruwys T, Dingle GA, Haslam C, Haslam SA, Jetten J, Morton TA (2013). Social group memberships protect against future depression, alleviate depression symptoms and prevent depression relapse. Social Science & Medicine 98, 179–186. 24331897 10.1016/j.socscimed.2013.09.013

[ref17] Dam J (2006). Insight in schizophrenia: a review. Nordic Journal of Psychiatry 60, 114–120. 16635929 10.1080/08039480600600185

[ref18] Ellemers N, Spears R, Doosje B (2002). Self and social identity. Annual Review of Psychology 53, 161–186. 10.1146/annurev.psych.53.100901.135228 11752483

[ref19] Fennig S, Everett E, Bromet EJ, Jandorf L, Fennig SR, Tanenberg-Karant M, Craig TJ (1996). Insight in first-admission psychotic patients. Schizophrenia Research 22, 257–263. 9000323 10.1016/s0920-9964(96)00077-1

[ref20] Giugiario M, Crivelli B, Mingrone C, Montemagni C, Scalese M, Sigaudo M, Rocca G, Rocca P (2012). Cognitive function and competitive employment in schizophrenia: relative contribution of insight and psychopathology. Social Psychiatry and Psychiatric Epidemiology 47, 553–561. 21451974 10.1007/s00127-011-0367-7

[ref21] Gómez-de-Regil L, Kwapil TR, Blanqué JM, Vainer E, Montoro M, Barrantes-Vidal N (2010). Predictors of outcome in the early course of first-episode psychosis. The European Journal of Psychiatry 24, 87–97.

[ref22] Haslam C, Holme A, Haslam SA, Iyer A, Jetten J, Williams WH (2008). Maintaining group memberships: social identity continuity predicts well-being after stroke. Neuropsychological Rehabilitation 18, 671–691. 18924001 10.1080/09602010701643449

[ref23] Haslam SA, Jetten J, Postmes T, Haslam C (2009). Social identity, health and well-being: an emerging agenda for applied psychology. Applied Psychology 58, 1–23.

[ref24] Henriksen MG, Parnas J (2014). Self-disorders and schizophrenia: a phenomenological reappraisal of poor insight and noncompliance. Schizophrenia Bulletin 40, 542–547. 23798710 10.1093/schbul/sbt087 PMC3984518

[ref25] Jones SH, Thornicroft G, Coffey M, Dunn G (1995). A brief mental health outcome scale-reliability and validity of the Global Assessment of Functioning (GAF). British Journal of Psychiatry 166, 654–659. 7620753 10.1192/bjp.166.5.654

[ref26] Karow A, Pajonk F-G, Reimer J, Hirdes F, Osterwald C, Naber D, Moritz S (2008). The dilemma of insight into illness in schizophrenia: self- and expert-rated insight and quality of life. European Archives of Psychiatry and Clinical Neuroscience 258, 152–159. 18000637 10.1007/s00406-007-0768-5

[ref27] Kay SR, Fiszbein A, Opfer LA (1987). The Positive and Negative Syndrome Scale (PANSS) for Schizophrenia. Schizophrenia Bulletin 13, 261–276. 3616518 10.1093/schbul/13.2.261

[ref28] Kravetz S, Faust M, David M (2000). Accepting the mental illness label, perceived control over the illness, and quality of life. Psychiatric Rehabilitation Journal 23, 323–332.

[ref29] Kreft I, De Leeuw J (1998). Introducing Multilevel Modelling. Sage: London.

[ref30] Lauber C, Nordt C, Falcato L, Rössler W (2000). Public acceptance of restrictions on mentally ill people. Acta Psychiatrica Scandinavica 102, 26–32. 11261636 10.1034/j.1600-0447.2000.00005.x

[ref31] Lincoln TM, Lüllmann E, Rief W (2007). Correlates and long-term consequences of poor insight in patients with schizophrenia. A systematic review. Schizophrenia Bulletin 33, 1324–1342. 17289653 10.1093/schbul/sbm002 PMC2779879

[ref32] Lysaker PH, Roe D, Yanos PT (2007). Toward understanding the insight paradox: internalized stigma moderates the association between insight and social functioning, hope, and self-esteem among people with schizophrenia spectrum disorders. Schizophrenia Bulletin 33, 192–199. 16894025 10.1093/schbul/sbl016 PMC2012366

[ref33] McEvoy JP, Johnson J, Perkins D, Lieberman JA, Hamer RM, Keefe RSE, Tohen M, Glick ID, Sharma T (2006). Insight in first-episode psychosis. Psychological Medicine 36, 1385–1393. 16740175 10.1017/S0033291706007793

[ref34] Mintz AR, Addington J, Addington D (2004). Insight in early psychosis: a 1-year follow-up. Schizophrenia Research 67, 213–217. 14984880 10.1016/S0920-9964(03)00047-1

[ref35] Mohamed S, Rosenheck R, McEvoy J, Swartz M, Stroup S, Lieberman JA (2009). Cross-sectional and longitudinal relationships between insight and attitudes toward medication and clinical outcomes in chronic schizophrenia. Schizophrenia Bulletin 35, 336–346. 18586692 10.1093/schbul/sbn067 PMC2659303

[ref36] O'Connor JA, Wiffen B, DiForti M, Ferraro L, Joseph C, Kolliakou A, Bonaccorso S, Murray RM, David AS (2013). Neuropsychological, clinical and cognitive insight predictors of outcome in a first episode psychosis study. Schizophrenia Research 149, 70–76. 23815972 10.1016/j.schres.2013.06.005

[ref37] Overall JE, Gorham DR (1962). The brief psychiatric rating scale. Psychological Reports 10, 799–812.

[ref38] Parellada M, Fraguas D, Bombín I, Otero S, Castro-Fornieles J, Baeza I, Gonzalez-Pinto A, Graell M, Soutullo C, Paya B, Arango C (2009). Insight correlates in child- and adolescent-onset first episodes of psychosis: results from the CAFEPS study. Psychological Medicine 39, 1433–1445. 19091160 10.1017/S0033291708004868

[ref40] Pijnenborg GHM, van Donkersgoed RJM, David AS, Aleman A (2013). Changes in insight during treatment for psychotic disorders: a meta-analysis. Schizophrenia Research 144, 109–117. 23305612 10.1016/j.schres.2012.11.018

[ref41] Raffard S, Fond G, Brittner M, Bortolon C, Macgregor A, Boulenger J-P, Gely-Nargeot M-C, Capdevielle D (2013). Cognitive insight as an indicator of competence to consent to treatment in schizophrenia. Schizophrenia Research 144, 118–121. 23313358 10.1016/j.schres.2012.12.011

[ref42] Rasbah J, Steele F, Browne WJ, Goldstein H (2009). A User's Guide to MLwiN. University of Bristol: Center for Multilevel Modelling (http://www.bristol.ac.uk/media-library/sites/cmm/migrated/documents/manual-print.pdf). Accessed 16 October 2015.

[ref43] Rinaldi M, Killackey E, Smith J, Shepherd G, Singh SP, Craig T (2010). First episode psychosis and employment: a review. International Review of Psychiatry 22, 148–162. 20504055 10.3109/09540261003661825

[ref44] Saeedi H, Addington J, Addington D (2007). The association of insight with psychotic symptoms, depression, and cognition in early psychosis: a 3-year follow-up. Schizophrenia Research 89, 123–128. 17097272 10.1016/j.schres.2006.09.018

[ref45] Tajfel H, Turner JC (1986). The social identity theory of intergroup behaviour In Psychology of Intergroup Relations (ed. S. Worchel and W. G. Austin), pp. 7–24. Nelson-Hall: Chicago.

[ref46] Thompson KN, McGorry PD, Harrigan SM (2001). Reduced awareness of illness in first-episode psychosis. Comprehensive Psychiatry 42, 498–503. 11704943 10.1053/comp.2001.27900

[ref47] Thornicroft G, Brohan E, Rose D, Sartorius N, Leese M (2009). Global pattern of experienced and anticipated discrimination against people with schizophrenia: a cross-sectional survey. The Lancet 373, 408–415. 10.1016/S0140-6736(08)61817-6 19162314

[ref48] Tranulis C, Lepage M, Malla A (2008). Insight in first episode psychosis: who is measuring what? Early Intervention in Psychiatry 2, 34–41. 21352129 10.1111/j.1751-7893.2007.00054.x

[ref49] van Baars AWB, Wierdsma AI, Hengeveld MW, Mulder CL (2013). Improved insight affects social outcomes in involuntarily committed psychotic patients: a longitudinal study in the Netherlands. Comprehensive Psychiatry 54, 873–879. 23618608 10.1016/j.comppsych.2013.03.016

[ref50] WHOQOL Group (1995). WHOQOL Group, The World Health Organization Quality of Life Assessment: position Paper from the World Health Organization. Social Science and Medicine 41, 1403–1409. 8560308 10.1016/0277-9536(95)00112-k

[ref51] Williams CC (2008). Insight, stigma, and post-diagnosis identities in schizophrenia. Psychiatry: Interpersonal and Biological Processes 71, 246–256. 10.1521/psyc.2008.71.3.246 18834275

[ref52] Yung AR, Yuen HP, McGorry PD, Phillips LJ, Kelly D, Dell'Olio M, Francey SM, Cosgrave E, Killackey E, Stanford C, Godfrey K, Buckby J (2005). Mapping the onset of psychosis: the comprehensive assessment of at-risk mental states. The Australian and New Zealand Journal of Psychiatry 39, 964–971. 16343296 10.1080/j.1440-1614.2005.01714.x

